# Metapopulation dynamics over 25 years of a beetle, *Osmoderma eremita*, inhabiting hollow oaks

**DOI:** 10.1007/s00442-020-04794-7

**Published:** 2020-11-07

**Authors:** Ly Lindman, Mattias C. Larsson, Kajsa Mellbrand, Glenn P. Svensson, Jonas Hedin, Olov Tranberg, Thomas Ranius

**Affiliations:** 1 Department of Ecology, Swedish University of Agricultural Sciences, Box 7044, 750 07 Uppsala, Sweden; 2 Department of Plant Protection Biology, Swedish University of Agricultural Sciences, Box 102, 230 53 Alnarp, Sweden; 3 County Administrative Board of Södermanland County, 611 86 Nyköping, Sweden; 4 Department of Biology, Lund University, 223 62 Lund, Sweden; 5 County Administrative Board of Kalmar County, 391 86 Kalmar, Sweden; 6 Department of Wildlife, Fish and Environmental Studies, Swedish University of Agricultural Sciences, 901 83 Umeå, Sweden

**Keywords:** Population size, Long-term data, Capture-mark-recapture, Colonisation, Extinction

## Abstract

*Osmoderma eremita* is a species of beetle that inhabits hollows in ancient trees, which is a habitat that has decreased significantly during the last century. In southeastern Sweden, we studied the metapopulation dynamics of this beetle over a 25 year period, using capture-mark-recapture. The metapopulation size had been rather stable over time, but in most of the individual trees there had been a positive or negative trend in population development. The probability of colonisation was higher in well-connected trees with characteristics reflecting earlier successional stages, and the probability of extinction higher in trees with larger diameter (i.e. in later successional stages), which is expected from a habitat-tracking metapopulation. The annual tree mortality and fall rates (1.1% and 0.4%, respectively) are lower than the colonisation and extinction rates (5–7%), indicating that some of the metapopulation dynamics are due to the habitat dynamics, but many colonisations and extinctions take place for other reasons, such as stochastic events in small populations. The studied metapopulation occurs in an area with a high density of hollow oaks and where the oak pastures are still managed by grazing. In stands with fewer than ten suitable trees, the long-term extinction risk may be considerable, since only a small proportion of all hollow trees harbours large populations, and the population size in trees may change considerably during a decade.

**Electronic supplementary material:**

The online version of this article (10.1007/s00442-020-04794-7) contains supplementary material, which is available to authorized users.

## Introduction

Metapopulation theory provides a framework for studying population dynamics in fragmented landscapes (Ovaskainen and Hanski 2004), and is thus relevant in species conservation (Hanski 1998). It has been recognised that metapopulations differ in their pattern of local extinctions (Harrison and Taylor 1997). In a classic metapopulation, all local populations face the risk of extinction due to stochastic events in patches that remain suitable (Hanski 1994). In contrast, in a habitat-tracking metapopulation, local populations often go extinct due to habitat deterioration (Thomas 1994). Furthermore, mainland–island metapopulations are characterized by a large difference in extinction risk amongst populations, with some local populations (inhabiting ‘mainlands’) having almost no extinction risk (Harrison and Taylor 1997). Most empirical studies on metapopulation ecology have been conducted on species with a rapid turnover, i.e. for which most local populations are exposed to a considerable extinction risk (Hanski 1999), such as many butterfly species (Hanski et al. 1994; Hanski and Thomas 1994; Cabeza et al. 2010). For such species, their dynamics can mostly be revealed even in short-term studies. Studies examining species with slower dynamics would increase our understanding of the relevance of metapopulation theory for a broader range of organism groups, but that would require long-term assessments.

Ancient trees constitute a habitat that is decreasing significantly worldwide (Lindenmayer et al. 2014). With the aging of trees, hollows are often formed (Ranius et al. 2009a). Inside tree hollows, wood mould (which is a mixture of decayed wood, fungi, remnants and frass from insects and other animals) supports a specialized invertebrate fauna (Siitonen and Ranius 2015). Hollows with wood mould represent a long-lived habitat that may last over many decades or even more than a century (Ranius et al. 2009b).

*Osmoderma eremita* is a beetle species that has been studied more than any other organism inhabiting hollows with wood mould. According to simulations based on field data from a few years, its metapopulation dynamics are slow, implying that it may take centuries from a decrease in the number of hollow trees until the *O. eremita* population finally goes extinct (Ranius and Hedin 2004). This is due to the low dispersal capability of the beetle (Ranius and Hedin 2001; Chiari et al. 2012) and the long lifetime of the hollow trees (Ranius et al. 2009b). Therefore, in order to assess the species’ metapopulation dynamics, long-term observational studies are needed.

In this study we explore the metapopulation dynamics of *O. eremita* over 25 years (1995–2019) in an area with a high density of old oaks in southeastern Sweden. Such a long study period is rare amongst metapopulation studies (see, however, several butterfly studies, e.g. Van Strien et al. 2011; Ojanen et al. 2013; Johansson et al. 2017). More specifically, we assessed the fluctuations and trends in the population size in individual trees and the whole metapopulation. The metapopulation turnover was assessed on the basis of colonisation and extinction rates. When doing so, we tested three hypotheses:

i The metapopulation is a mainland–island type, resulting in large differences in population size between trees, with the same trees having the largest populations over time (i.e. constituting the mainlands).
ii The metapopulation is habitat-tracking, resulting in the probability of colonisation and extinction increasing with tree characteristics reflecting early or late successional stages, respectively.
iii The local extinction rate is both driven by habitat dynamics and by internal population dynamics, resulting in local extinction rates being higher than tree mortality and fall rate.

## Material and methods

### Study species

*O. eremita* is a 3-cm long Scarabaeid beetle, which has been allocated the highest conservation priority in the EU’s Habitat Directive Annex II and IV (European Council 1992). The larvae develop exclusively inside hollow trees (Ranius et al. 2005). In Sweden, *O. eremita* primarily inhabits oaks (*Quercus robur*) in wooded pastures. Adults emerge in July and live for a few weeks. They mainly remain in the same tree hollow (Ranius and Hedin 2001), and do not feed. The total development time of *O. eremita* is usually 3 or 4 years (Ranius et al. 2005).

### Study area and design

**Fig. 1:**
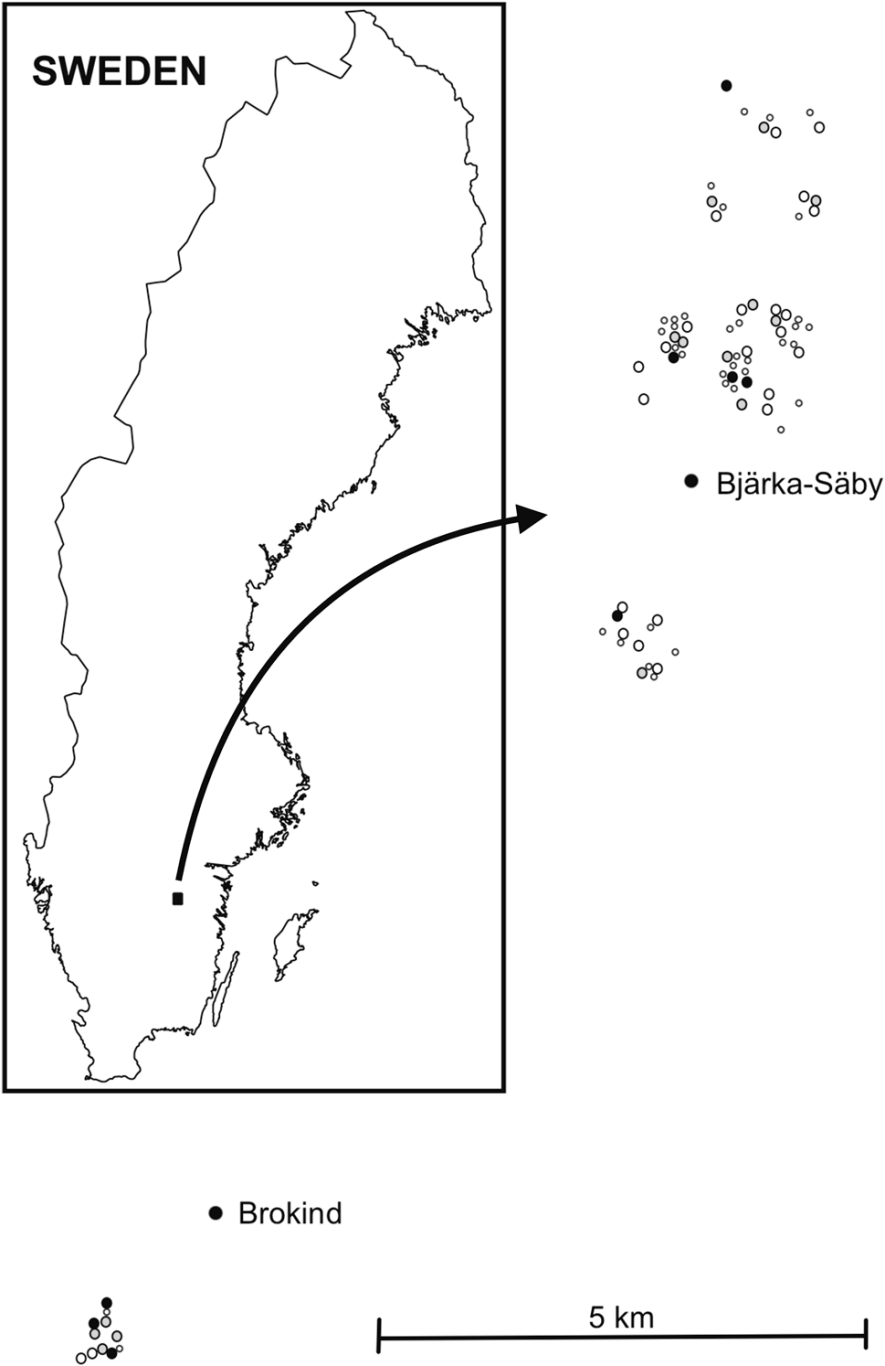
Location of the trees in Bjärka-Säby and Brokind (Östergötland, Sweden), where *Osmoderma eremita* has been studied. The different symbols indicate the estimated population size per tree in 2019: black filled dots mark > 10 *O. eremita* individuals, grey filled dots mark < 10 individuals, black open circles trees where no individuals were found, and small grey open dots indicate hollow trees not surveyed

This study was conducted in two areas in southeastern Sweden which were chosen because of their high density of hollow oaks: Bjärka-Säby (58°16ʹ N, 15°46ʹ E), and Brokind (58°12ʹ N, 15°40ʹ E). The two areas include a total of approximately 200 hectares. Sampling of trees (Fig. 1) for adult *O. eremita* was undertaken in the following years: 1995–2002, 2005, 2006, 2008, 2015, and 2019. During the first three years we sampled only in the core areas of Bjärka-Säby, representing approximately 30% of the trees included in 1998. In 1998 and thereafter, a larger area was studied, and in 1998 all hollow trees with cavities in which it was possible for a pitfall trap to be retained were selected for trapping. In practical terms, this meant that we excluded trees that had hollows higher than 5 m above the ground (length of the ladder), were too narrow to access, had too little accessible wood mould in which to place a pitfall trap or where the wood mould was inaccessible (too far from the entrance hole to be reached). In each hollow we placed a pitfall trap (an empty jar) with the opening at the level of the wood mould surface (Ranius 2001). If possible, traps with a top diameter of 7 cm were used, although in narrow hollows they were 5–6 cm wide. The traps were emptied every second day (or, during the first three years, every day) from early or mid-July to mid-August. Each trapped beetle was given an individual mark on the elytra and thereafter released on the surface of the wood mould.

### Population size

For estimating population size from capture-mark-recapture data, we used Craig’s (1953) model: log *N –* log (*N – r*) = *s* × *N*^−1^, in which *N* is the population size, *r* is the number of captured individuals, and *s* is the number of captures. Recently, different models based on the Jolly–Seber method (Jolly 1965) have frequently been used for population size estimates (Schwarz 2001; Madon et al. 2011; Marschalek 2020). With the Jolly–Seber method it is possible to estimate the population size for certain points in time, whilst with Craig’s model we only obtain the size of the population that have been present during the whole season, which is needed in the present study. In a previous analysis, our capture-mark-recapture data over 4 years was analysed with both Craig’s model and the Jolly–Seber methods, and it resulted in very similar estimates of the population size (Ranius 2001). Also for other species, Craig’s model has been found to provide fairly accurate estimates of population size (Matter and Roland 2004). Here we used only Craig’s model, since it requires less data. However, even with this method it was not possible to calculate the population size for each tree and year separately, as many trees had no recapture events. Therefore, we divided the trees and years into two groups: (i) trees and years with > 10 captured individuals, and (ii) trees and years with ≤ 10 captured individuals. In the first case we used Craig’s model for each tree and year separately. In the second case, we used Craig’s model to estimate the total population size for each year. This estimate was divided by the number of captured individuals. The population size in each tree and year was estimated by multiplying this rate by the number of captured individuals in each tree. Standard error values were obtained for each population size estimate using Craig’s method. Thus, it was estimated only for trees and years with > 10 captured individuals (see Online Resource 1).

When describing the development of the total metapopulation size, we used the average population size per sampled tree, since the number of trees surveyed each year was not the same. The coefficient of variation (CV) was used as a measure of temporal variability. CV = (SD over time) × (mean over time)^−1^ was calculated both for the average population size per sampled tree and for the populations in individual trees.

### Colonisation rate and extinction risk

We observed colonisations and extinctions by comparing presence/absence over three sequential sampling years at a time. Our main way to do this was based on assumptions derived from the species’ biology (with a developmental time of 3 or 4 years) and the risk of observing false absences or presences. As a sensitivity test we compared the colonisation rate and extinction risk when applying other assumptions (observing colonisations and extinctions by comparing presence/absence over one and two sequential sampling years at the time). In our main approach, at least three adults found per year was regarded as a presence and no adults found was regarded as an absence. When one or two adults were found, we assumed that the status (presence or absence) was the same as the year before (or for the first monitoring the year later), because one or two adults have been found in certain years in trees where no more adults were ever recorded. This was done to avoid false presences due to immigration to trees where larvae never developed. Only when beetles were recorded as absent in three sequential sampling years was the population considered absent, since adults can be absent from a tree even though the species still exists as larvae. When sampling did not occur every year, sequential sampling years were taken into account even though the time between the sampling was greater. Therefore, it was also possible to observe colonisation and extinction rates for the latter part of the period of study, when sampling did not occur every year.

We analysed the probability of colonisation and extinction with tree characteristics reflecting habitat amount, microclimate, and successional stage (Table 1), using binomial logistic regression with tree ID as a random factor. Due to the relatively high number of potential predictors (*k* = 9), we performed a first selection of the variables, analysing one variable at a time, using second-order Akaike’s information criterion corrected for small sample size [AICc; R package *AICcmodavg* (Mazerolle 2019)], as recommended when *N* (sample size) × *k* (number of predictors)^−1^ < 40 (Burnham and Anderson 2002). We selected all variables that decreased the AICc value in comparison to the null model. After that we built multivariable models by testing all possible combinations of selected variables together with the covariate number of possible occasions using the R package *MuMIn* (Bartoń 2019). Models were ranked using the difference between their AICc score and the score of the best-fitting model (ΔAICc_*i*_ = AICc_*i*_ – AICc_min_); we considered models with ΔAICc < 2 plausible (Burnham and Anderson 2002).

**Table 1:** Tree variables used in the analyses

Variable	Definition	Year of measurement	Unit
Canopy coverage	Percentage of a 2 m wide zone around tree crown shaded by other tree crowns. Classes < 25 (0), 25–75 (1), > 75% (2)	2001, 2019	nr
Direction of entrance	Horizontal direction of entrance hole (angle)	2019	nr
Entrance height	Distance from the ground to the lower part of the entrance hole	2019	cm
Entrance size	Area of entrance hole	2019	cm^2^
Alive/dead	Whether the tree is alive (1) or not (0)	2001, 2019	nr
Tree age	Age estimated from coring	2006	years
Diameter	Calculated from the stem circumference at breast height (1.3 m)	2001, 2019	cm
Wood mould volume	Approximate wood mould volume in tree hollow	2001, 2019	dm^3^
Connectivity	Value expressing a potential for immigration	2019	m
No of possible occasions	How many years’ colonisations or extinctions it was possible to observe	2019	nr

For details about measurement of entrance size, wood mould volume, and connectivity, see Methods

Thereafter, we calculated colonisation rate and extinction risk for each year by dividing the number of trees in which colonisation or extinction events were observed by the number of trees where it was possible to observe these events. As a sensitivity analysis, we also tested to use (i) only one or (ii) two years of absence in a row, and only (iii) for three years in a row (which implies that when the sampling did not occur every year, observation of colonisations and extinctions was impossible).

### Tree characteristics

Tree characteristics, which have been previously tested in studies of *O. eremita* (Ranius and Nilsson 1997; Ranius and Jansson 2000; Ranius 2002, 2007), were measured in 2001 and 2019 (Table 1). *Diameter* data were collected in 2019, but for those trees not visited in 2019, we used data from 2001, and added 2 × 18 × (annual ring width measured at each location) (Ranius et al. 2009b). The *entrance size* and wood mould surface in the hollow were measured on the basis of the shape they most resembled (ellipse, circle, triangle, or rectangle). The wood mould surface area (*S*) and the depth of the cavity from the surface were used for calculating *wood mould volume* (*V* = 1/3 × depth of cavity × surface area *S*). In the analyses, we used the geometric average of the wood mould volumes measured in 2001 and 2019. When one of the values was missing, only the other one was used. *Direction of entrance* was categorized according to the sunpath diagram, taking account of the intensity of the sun’s radiation. We divided the circle (360º) into 24 sections which were numbered according to the distance from south (180º), resulting in 12 categories with 1 being the most southerly and 12 the most northerly direction. For *tree age*, we used values measured from cores collected in 2005 (see Ranius et al. 2009a) and added the number of years (14) that had passed.

We calculated* connectivity*, which according to metapopulation theory affects colonisation rate and extinction risk (Hanski 1998), using the following equation:

$$S_{i} = \mathop \sum \limits_{j = 1}^{n} p_{j } \exp \left( { - \alpha d_{ij} } \right), \;{\text{for all}}\; j \ne i,$$

where *p*_*j*_ is the average population size over time in all surveyed trees *j* (Ranius et al. 2010). The value of *α* was set to 60^−1^ based on the proportion of dispersing *O. eremita* individuals with dispersal ranges exceeding certain distances observed in previous dispersal studies (Ranius 2006). *d*_*ij*_ is the distance between trees *i* and *j* (in m). Comparing habitat variables *alive/dead* and *tree position*, measured in 2001 and 2019, allowed us to calculate tree mortality and fall rate over 18 years.

## Results

### Population size

**Fig. 2:**
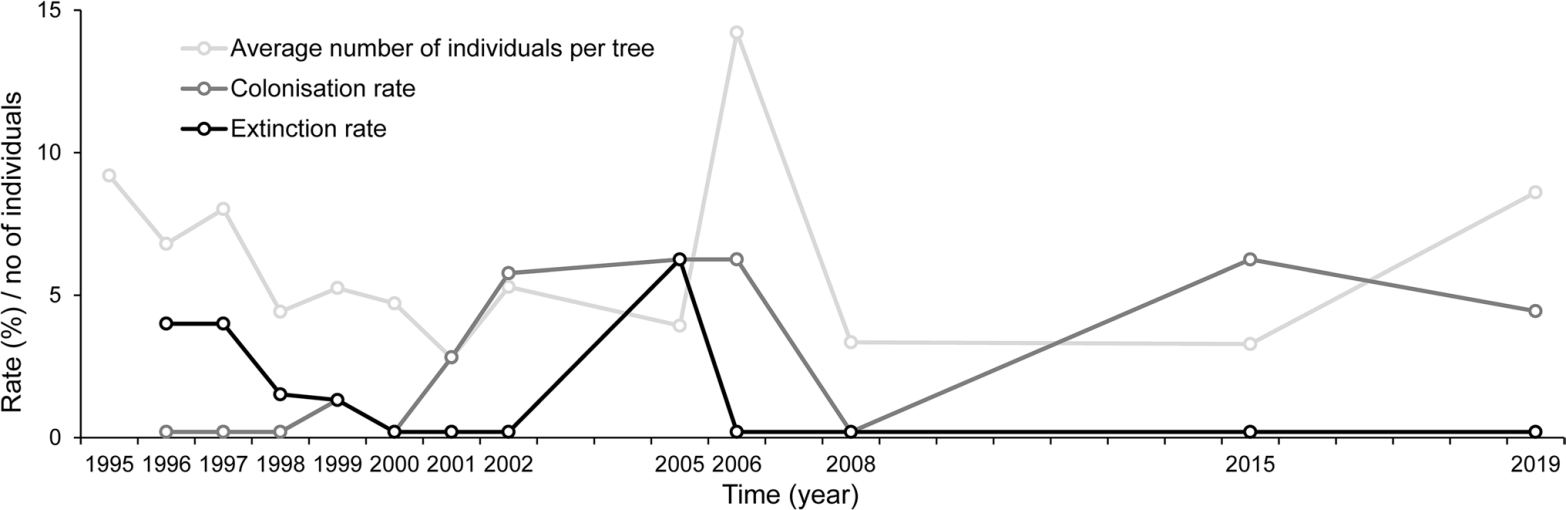
Average number of individuals per tree (light grey symbols), colonisation rate (%, dark grey symbols), and extinction risk (%, black symbols) of *Osmoderma eremita* from 1995 to 2019

*O. eremita* was found in 39 (50%) of 78 sampled trees. The average population size per tree varied from 2.8 to 14.2 between years (Table 2), and from 0 to 103 between individual trees. The average population size per sampled tree was relatively stable over time (Fig. 2), whilst for individual trees the population size often changed considerably over time.

**Table 2:** Number of sampled trees, captures and captured *Osmoderma eremita* individuals, estimated population size and average number of individuals per tree from 1995 to 2019

Year	No. of sampled trees	Captures	Captured individuals	Estimated population size	Average population size per sampled tree
1995	25	283	165	227	9.2
1996	25	214	108	170	6.8
1997	25	311	140	198	8.0
1998	66	542	203	285	4.4
1999	76	422	244	390	5.3
2000	77	436	225	357	4.7
2001	71	206	129	196	2.8
2002	52	279	174	280	5.3
2005	32	212	100	120	3.9
2006	32	388	227	441	14.2
2008	26	202	76	87	3.3
2015	49	170	108	159	3.3
2019	48	500	267	389	8.6

**Fig. 3:**
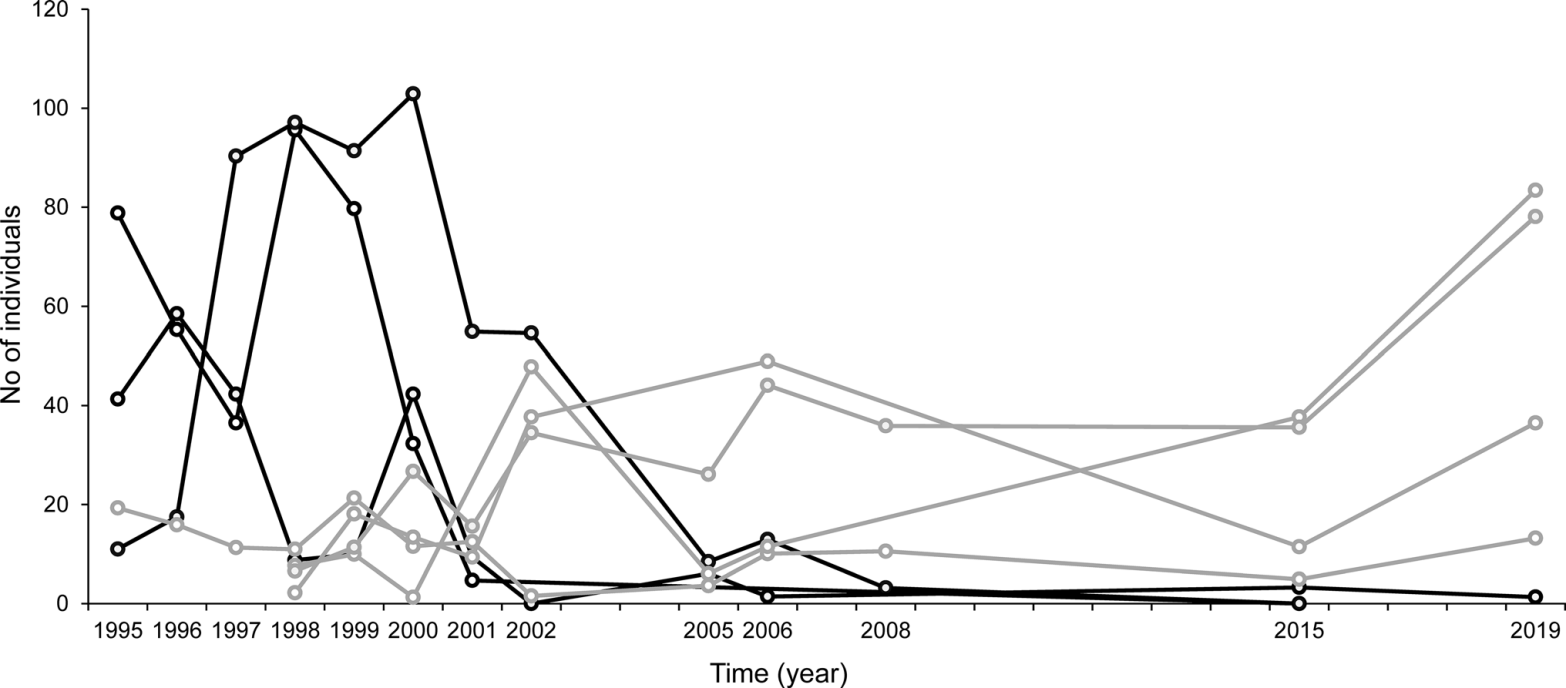
Changes in population size of *Osmoderma eremita* for the seven trees with the highest estimated population sizes (average over time) and at least eight trapping years over 25 years. Black lines represent trees recorded as being dead in 2001. Light grey lines represent trees that were alive in 2001

The temporal variability in population size was larger in individual trees than in the total metapopulation; CV for individual trees was on average 1.57 and for the average population size per sampled tree 0.52. For the seven trees with the largest populations, the value was 0.75 (Fig. 3). Amongst these seven trees, all trees with decreasing population sizes were recorded as being dead in 2001. The other four trees—one with stable population size and three with increasing population sizes—were alive in 2019.

### Colonisation rate, extinction risk and tree characteristics

The probability of colonisation increased with increased connectivity, and if trees were alive. In single models, the colonisation rate increased with decreasing amount of wood mould, entrance size, and canopy cover, but in other models with increasing entrance height and diameter. The probability of extinction increased with increasing tree diameter (Table 3), and in one model also with decreasing entrance height. When using other assumptions for when the species is present and absent, similar outcomes were obtained (see Online Resource 2).

**Table 3:** Plausible candidate models (ΔAIC < 2) explaining colonisations and extinctions of *O. eremita*

*N*	Int.	Parameter estimates								*k*	LogLik	ΔAICc	*w*_*i*_	$R_{{{\text{MF}}}}^{2}$
		Connectivity	Alive/dead	Wood mould volume	Entr. size	Entr. height	Canopy cover	Diameter	No of possible occ					
1. Colonisation														
50														
	0.13	0.032							− 0.34	4	− 26.22	0.00	0.18	0.12
	− 0.46		0.792						− 0.31	4	− 26.53	0.63	0.13	0.11
	0.56			− 0.008					− 0.33	4	− 26.57	0.71	0.13	0.10
	0.36				− 0.003				− 0.32	4	− 26.82	1.20	0.10	0.10
	0.21					0.001			− 0.35	4	− 26.82	1.21	0.10	0.10
	0.82						− 0.671		− 0.35	4	− 26.65	1.34	0.10	0.13
	− 0.02							0.002	− 0.33	4	− 26.92	1.41	0.10	0.09
	− 0.73	0.034	0.859						− 0.31	5	− 25.71	1.46	0.09	0.13
	0.38	0.031		− 0.008					− 0.35	5	− 25.83	1.71	0.08	0.13
2. Extinction														
20														
	− 4.70							0.037	− 0.55	4	− 4.92	0.00	0.69	0.51
	− 4.44					− 0.005		0.040	− 0.56	5	− 3.90	1.58	0.31	0.61

Tree ID as a random factor is included in all models. Models are ranked according to their second-order Akaike’s information criterion (AIC_c_). Sample size (*N*), intercept (Int.), number of parameters (*k*), model weight (*w*_*i*_) and McFadden’s *R*^2^ are reported

Colonisation rate and extinction risk, using our main approach (population absence recorded only when absent in three sequential sampling years), were 7.2% and 5.1%, respectively. They were relatively stable over time (Fig. 2). When population absence was considered to have occurred when the species was absent 2 years in a row, the rates were 6.6% and 8.1%, and for 3 years in a row (excluding years when sampling did not occur every year) 5.5% and 7.7%. The annual tree mortality was 1.1% (14 trees out of 68 died during the 18 years). The probability that a tree would fall was 0.4% per year (five trees of 74 had fallen during the 18 years).

## Discussion

Despite large changes in populations of *O. eremita* in individual trees, the metapopulation size was relatively stable over 25 years. Metapopulation dynamics seem to be important for this species, since it may persist at a metapopulation level even though the conditions and population sizes in individual trees are changing. Partly, the colonisations and local extinctions could also be the result of source-sink dynamics. However, it is less likely that this is an important process for this particular species, since the majority of adults remain in the same tree throughout their entire lifetime (Ranius 2001). Furthermore, in all trees with adults of *O. eremita*, frass of larvae is found (Ranius and Nilsson 1997), implying that observed adults are not only immigrants but at least at some occasions the tree has supported larval development.

*O. eremita* has been described as occurring in a mainland-island metapopulation, since there is wide variability in population size and thus also in population persistence between trees (Ranius 2007). However, since the largest populations, which seem to be “mainland populations”, do not remain that large over a longer period of time, they probably also suffer from a local extinction risk at a longer time scale (cf. Ranius 2007). Thus, our long-term survey reveals that *O. eremita* occurs in a mainland-island metapopulation to a lesser extent than previously thought.

High connectivity and that trees were alive were the two most important factors increasing the colonisation rate. The positive effect of connectivity on colonisation rate indicates that the immigration rate is higher for trees with higher connectivity. This effect is consistent with metapopulation theory (Ovaskainen and Hanski 2004) and with the low dispersal rate and range observed for the species (Ranius and Hedin 2001). This implies that the spatial distribution of habitat is important; suitable hollow trees are colonised by *O. eremita* and thus also contribute to metapopulation size to a greater extent if they are clustered rather than more isolated from each other. The importance of the spatial distribution of deadwood habitats has recently been questioned by Komonen and Müller (2018), but their conclusion was only based on maximum observed dispersal distances and interpretations of genetic patterns, whilst observations of colonisation patterns were not considered. Observations of colonisation patterns, as those in our study, give more direct evidence of the importance of connectivity for long-term population persistence in landscapes but require data from repeated surveys at multiple sites, which is rare.

According to several models, the colonisation rate increased with tree characteristics reflecting early decay stages (living trees, less wood mould, smaller entrance size; Ranius et al. 2009b). In other models, it was positively related with the diameter of the tree, the height between the ground and the entrance hole, or with a decreasing canopy cover, which all are variables that previously have been shown to increase the probability of occurrence of *O. eremita* (Chiari et al. 2012). In our study, *O. eremita* colonised only living trees. To some extent this supports the idea that dead trees are unsuitable for the species (Ranius et al. 2005). However, in one tree we observed the species had been persisting for more than a decade after the tree had fallen. Even though *O. eremita* tends to colonise hollow trees in early stages, the trees are still old; hollows starts to form when the oaks are 200 years (Ranius et al. 2009b).

Since it is impossible to trap beetles in hollows with narrow entrances, we have no *O. eremita* data from such trees. Trees with smaller entrances tend to be younger and contain smaller volumes of wood mould (Ranius et al. 2009a). Thus, these trees tend to be in an earlier successional stage and since population size tends to increase with the amount of wood mould (Ranius 2007), the *O. eremita* populations tend to be smaller in comparison to trees we studied. Thus, our study may not reflect the dynamics in all trees, but still it includes the trees most important for the metapopulation dynamics.

The probability of extinction increased with tree diameter and decreased with entrance height. The relationship with tree diameter reflects that the extinction risks increase during the successional development of the hollow trees. The tendency to colonise trees in earlier successional stages and become extinct in later stages is consistent with expectations for a habitat-tracking metapopulation (Thomas 1994). According to Thomas (1994), local colonisations and extinctions in metapopulations of rare and declining species are often consequences of successional changes of the habitat. This has previously been found for another wood-dependent beetle species, associated with burned forest habitats (Ranius et al. 2014).

Similar to the results presented by Drobyshev et al. (2008), the annual mortality of oaks in our study was 1.1%. The observed colonisation and extinction rates were higher (5–7%), indicating that whilst some of the metapopulation dynamics are due to the habitat dynamics, many colonisations and extinctions occur for other reasons. This means that *O. eremita* has more dynamic metapopulations than, for instance, specialised epiphytic lichens, which have very low extinction rates, once they have colonised an old oak tree (Johansson et al. 2012). Conclusively, our observations support the suggestion that beetle populations in hollow trees function as habitat-tracking metapopulations.

In our study area, the population size of *O. eremita* has been relatively stable over the last 25 years. The hollow oaks have historically been growing in a half-open landscape commonly used for hay-making and grazing. Today, around 70% of the studied trees are situated within pastures still grazed by cattle or in a deer park. Both on grazed and ungrazed land, shrubs and fast-growing small trees have been cut in the immediate vicinity of the old oaks. This is one reason why the proportion of sun-exposed trees has slightly increased over time (2001: 30% sun-exposed, 12% shaded; 2019: 42% sun-exposed, 21% shaded). Cessation of management would increase tree mortality and also be detrimental for many beetle species present (Ranius and Jansson 2000). Our study suggests that the conditions in the study area allow the persistence of *O. eremita*. However, the area is amongst only a few with a high density of hollow oaks, whilst the majority of sites with *O. eremita* are much smaller (Ranius et al. 2005), and in such sites the risk of extinction may be substantial. This is because, at such sites, the populations are probably often dependent on one or a few trees that harbour a large number of individuals. As a result, the long-term extinction risk may be elevated, since the population size may change considerably in individual trees within a decade.

### Implications for conservation

Our study shows that successional development and spatial connectivity of the habitat are the two main factors to consider in the conservation of *O. eremita*. In areas with numerous ancient trees there is often a comparatively low number of young trees (Miklín and Čížek 2014; Ranius et al. 2009a), indicating a potential decrease in ancient trees in the future (Gibbons et al. 2008; Miklín and Čížek 2014). For maintaining *O. eremita* metapopulations in the long term, existing hollow trees should be preserved and, if possible, their life-time prolonged (Keymer et al. 2000). New hollow trees should be established, and as the development of a hollow takes a very long time, perhaps this development can be accelerated by damaging younger trees (‘veteranisation’) (Siitonen and Ranius 2015). It may be done through activities like pollarding (Šebek et al. 2013), pruning of branches (Avilés 2019) and injuring the trees (Gibbons et al. 2000) through, for instance burning its base, boring holes, or hitting the trunk with a sledgehammer (Bengtsson et al. 2015). The spatial location of both existing and new trees is important to consider, since the probability of *O. eremita* colonising trees is higher if they are situated closer to existing populations.

## Electronic supplementary material

Below is the link to the electronic supplementary material.

Supplementary file1 (PDF 932 KB)

Supplementary file2 (PDF 447 KB)

## Data Availability

The datasets generated and/or analysed during the current study are available from the corresponding author on reasonable request.

## References

[CR1] Avilés JM (2019). Pruning promotes the formation of an insufficient number of cavities for hollow-dependent birds in Iberian Holm-oak dehesas. For Ecol Manag.

[CR2] Bartoń K (2019) MuMIn: multi-model inference. R package version 1.43.15. The Comprehensive R Archive Network (CRAN), Vienna

[CR3] Bengtsson V, Niklasson M, Hedin J (2015). Tree veteranisation—using tools instead of time. Conserv Land Manag.

[CR4] Burnham KP, Anderson DR (2002). Model selection and multimodel inference: a practical information-theoretic approach.

[CR5] Cabeza M, Arponen A, Jäättelä L, Kujala H, Van Teeffelen A, Hanski I (2010). Conservation planning with insects at three different spatial scales. Ecography.

[CR6] Chiari S, Carpaneto GM, Zauli A, Marini L, Audisio P, Ranius T (2012). Habitat of an endangered saproxylic beetle, *Osmoderma eremita*, in Mediterranean woodlands. Écoscience.

[CR7] European Council (1992). Council Directive 92/43/EEC of 21 May 1992 on the conservation of natural habitats and of wild fauna and flora. Off J Eur Communities.

[CR8] Craig CC (1953). On the utilization of marked specimens in estimating populations of flying insects. Biometrika.

[CR9] Drobyshev I, Niklasson M, Linderson H, Sonesson K, Karlsson M, Nilsson SG, Lannér J (2008). Lifespan and mortality of old oaks—combining empirical and modelling approaches to support their management in Southern Sweden. Ann For Sci.

[CR10] Gibbons P, Lindenmayer DB, Barry SC, Tanton MT (2000). Hollow formation in eucalypts from temperate forests in southeastern Australia. Pac Conserv Biol.

[CR11] Gibbons P, Lindenmayer DB, Fischer J, Manning AD, Weinberg A, Seddon J, Ryan P, Barrett G (2008). The future of scattered trees in agricultural landscapes. Conserv Biol.

[CR12] Hanski I (1994). A practical model of metapopulation dynamics. J Anim Ecol.

[CR13] Hanski I (1998). Metapopulation dynamics. Nature.

[CR14] Hanski I (1999). Metapopulation ecology.

[CR15] Hanski I, Thomas CD (1994). Metapopulation dynamics and conservation: a spatially explicit model applied to butterflies. Biol Cons.

[CR16] Hanski I, Kuussaari M, Nieminen M (1994). Metapopulation structure and migration in the butterfly *Melitaea cinxia*. Ecology.

[CR17] Harrison S, Taylor AD (1997) Empirical evidence for metapopulation dynamics. In: Hanski I, Gilpin ME (eds) Metapopulation biology. Ecology, genetics, and evolution. Academic Press, San Diego, pp 27–42. 10.1016/B978-012323445-2/50004-3L.558

[CR18] Johansson V, Ranius T, Snäll T (2012). Epiphyte metapopulation dynamics are explained by species traits, connectivity, and patch dynamics. Ecology.

[CR19] Johansson V, Knape J, Franzén M (2017). Population dynamics and future persistence of the clouded Apollo butterfly in southern Scandinavia: the importance of low intensity grazing and creation of habitat patches. Biol Cons.

[CR20] Jolly GM (1965). Explicit estimates from capture-recapture data with both death and immigration-stochastic model. Biometrika.

[CR21] Keymer JE, Marquet PA, Velasco-Hernández JX, Levin SA (2000). extinction thresholds and metapopulation persistence in dynamic landscapes. Am Nat.

[CR22] Komonen A, Müller J (2018). Dispersal ecology of deadwood organisms and connectivity conservation. Conserv Biol.

[CR23] Lindenmayer DB, Laurance WF, Franklin JF, Likens GE, Banks SC, Blanchard W, Gibbons P, Ikin K, Blair D, McBurney L, Manning AD, Stein JAR (2014). New policies for old trees: averting a global crisis in a keystone ecological structure. Conserv Lett.

[CR24] Madon B, Gimenez O, McArdle B, Baker CS, Garrigue C (2011). A new method for estimating animal abundance with two sources of data in capture–recapture studies. Methods Ecol Evol.

[CR25] Marschalek DA (2020). Sex-biased recapture rates present challenges to quantifying population sizes and dispersal behavior of the regal fritillary butterfly (*Speyeria idalia*). J Insect Conserv.

[CR26] Matter SF, Roland J (2004). Relationships among population estimation techniques: an examination for *Parnassius smintheus* Doubleday (Papilionidae). J Lepid Soc.

[CR27] Mazerolle MJ (2019) AICcmodavg: model selection and multimodel inference based on (Q) AIC(c). R package version 2.2–2. The Comprehensive R Archive Network (CRAN), Vienna

[CR28] Miklín J, Čížek L (2014). Erasing a European biodiversity hot-spot: open woodlands, veteran trees and mature forests succumb to forestry intensification, succession, and logging in a UNESCO Biosphere Reserve. J Nat Conserv.

[CR30] Ojanen SP, Nieminen M, Meyke E, Pöyry J, Hanski I (2013). Long-term metapopulation study of the Glanville fritillary butterfly (*Melitaea cinxia*): survey methods, data management, and long-term population trends. Ecol Evol.

[CR31] Ovaskainen O, Hanski I (2004). From individual behavior to metapopulation dynamics: unifying the patchy population and classic metapopulation models. Am Nat.

[CR32] Ranius T (2001). Constancy and asynchrony of *Osmoderma eremita* populations in tree hollows. Oecologia.

[CR33] Ranius T (2002). *Osmoderma eremita* as an indicator of species richness of beetles in tree hollows. Biodivers Conserv.

[CR34] Ranius T (2006). Measuring the dispersal of saproxylic insects: a key characteristic for their conservation. Popul Ecol.

[CR35] Ranius T (2007). Extinction risks in metapopulations of a beetle inhabiting hollow trees predicted from time series. Ecography.

[CR36] Ranius T, Hedin J (2001). The dispersal rate of a beetle, *Osmoderma eremita*, living in tree hollows. Oecologia.

[CR37] Ranius T, Jansson N (2000). The influence of forest regrowth, original canopy cover and tree size on saproxylic beetles associated with old oaks. Biol Cons.

[CR38] Ranius T, Nilsson SG (1997). Habitat of *Osmoderma eremita* Scop. (Coleoptera: Scarabaeidae), a beetle living in hollow trees. J Insect Conserv.

[CR39] Ranius T, Hedin J, Akçaya HR (2004). Hermit beetle (*Osmoderma eremita*) in a fragmented landscape: predicting occupancy patterns. Species conservation and management: case studies.

[CR40] Ranius T, Aguado LO, Antonsson K, Audisio P, Ballerio A, Carpaneto GM, Chobot K, Gjurašin B, Hanssen O, Huijbregts H, Lakatos F, Martin O, Neculiseanu Z, Nikitsky NB, Paill W, Pirnat A, Rizun V, Ruicănescu A, Stegner J, Süda I, Szwałko P, Tamutis V, Telnov D, Tsinkevich V, Versteirt V, Vignon V, Vögeli M, Zach P (2005). *Osmoderma eremita* (Coleoptera: Scarabaeidae: Cetoniinae) in Europe. Anim Biodivers Conserv.

[CR41] Ranius T, Niklasson M, Berg N (2009). Development of tree hollows in pedunculate oak (*Quercus robur*). For Ecol Manag.

[CR42] Ranius T, Svensson GP, Berg N, Niklasson M, Larsson MC (2009). The successional change of hollow oaks affects their suitability for an inhabiting beetle, *Osmoderma eremita*. Ann Zool Fenn.

[CR43] Ranius T, Johansson V, Fahrig L (2010). A comparison of patch connectivity measures using data on invertebrates in hollow oaks. Ecography.

[CR44] Ranius T, Bohman P, Hedgren O, Wikars L-O, Caruso A (2014). metapopulation dynamics of a beetle species confined to burned forest sites in a managed forest region. Ecography.

[CR45] Schwarz CJ (2001). The Jolly–Seber model: more than just abundance. J Agric Biol Environ Stat.

[CR46] Šebek P, Altman J, Platek M, Cizek L (2013). Is active management the key to the conservation of saproxylic biodiversity? Pollarding promotes the formation of tree hollows. PLoS ONE.

[CR47] Siitonen J, Ranius T, Kirby KJ, Watkins C (2015). The importance of veteran trees for saproxylic insects. (2015) Europe’s changing woods and forests: from wildwood to managed landscapes.

[CR48] Thomas CD (1994). Extinction, colonisation, and metapopulations—environmental tracking by rare species. Conserv Biol.

[CR49] Van Strien AJ, van Swaay CAM, Kéry M (2011). Metapopulation dynamics in the butterfly *Hipparchia semele* changed decades before occupancy declined in The Netherlands. Ecol Appl.

