# Supplementary material for: Metapopulation dynamics over 25 years of a beetle, Osmoderma eremita, inhabiting hollow oaks
Source: Oecologia. 2020 Nov 7;194(4):771–80. doi: 10.1007/s00442-020-04794-7 (PMC7683440; doi:10.1007/s00442-020-04794-7)
Supplement: Supplementary file 1 — Supplementary file1 (PDF 932 KB) [file 442_2020_4794_MOESM1_ESM.pdf]

Online Resource 1

Journal: Oecologia

Title: Metapopulation dynamics over 25 years of a beetle, *Osmoderma eremita*, inhabiting hollow oaks

Authors: Ly Lindman, Mattias C. Larsson, Kajsa Mellbrand, Glenn P. Svensson, Jonas Hedin, Olov Tranberg, Thomas Ranius

Corresponding author: L. Lindman, e-mail: Ly.Lindman@slu.se

Online Resource 1 Calculated *catchability* indices, number of captured individuals (*r*), number of captures (*s*), population sizes (N_C; for trees with ≤10 captured individuals) calculated with the probability rate of capturing an individual (Capture %), population sizes (N_{CR}; for trees with > 10 captures individuals) calculated with Craig´s model separately for each tree, and standard errors (*SE*) for population sizes for N_{CR} are presented for each tree for each monitored year. One continues table is divided by three for better fit on the sheet: 1995-1999, 2000-2006, and 2008-2019

Year	1995					1996					1997					1998					1999				
Capture %			1.37					1.99					1.11					1.18					1.52		
ID	r	s	N_C	N_{CR}	SE	r	s	N_C	N_{CR}	SE	r	s	N_C	N_{CR}	SE	r	s	N_C	N_{CR}	SE	r	s	N_C	N_{CR}	SE
1.1																0	0	0			1	1	1.5		
1.2	58	105		78.8	7.3	47	105		55.3	3.8	20	29		36.5	9.3	30	36		95.6	34.4	25	30		79.7	31.4
1.3	3	9	4.1			1	2	2.0			0	0	0			0	0	0			0	0	0		
1.4	1	1	1.4			0	0	0			0	0	0			0	0	0			11	19		15.6	3.7
1.5	2	2	2.7			0	0	0			0	0	0			0	0	0			1	1	1.5		
1.6																					0	0	0		
1.7																					15	45		15.9	1.1
1.8																					0	0	0		
1.9																					5	5	7.6		
1.10																0	0	0			0	0	0		
1.11																0	0	0			0	0	0		
1.13																									
2.1	11	13		11	6.9	15	34		17.5	2.1	78	180		90.3	4.6	94	335		97.1	1.9	66	117		91.4	8.3
2.2	0	0	0			0	0	0			2	7	2.2			0	0	0			0	0	0		
2.3	0	0	0			0	0	0			1	2	1.1			0	0	0			0	0	0		
2.4	0	0	0			0	0	0			0	0	0			0	0	0			0	0	0		
3.1	29	50		41.3	6.0	14	16		58.5	37.8	14	17		42.3	21.4	8	13	9.5			7	8	10.6		
3.2	5	10	6.8			0	0	0			2	11	2.2			4	29	4.7			5	13	7.6		
3.3	8	15	11.0			3	6	6.0			8	20	8.8			5	5	5.9			4	7	6.1		
3.4	14	25		19.3	3.8	14	34		15.9	1.7	11	41		11.3	0.6	10	27	11.8			15	26		21.3	4.3
3.5	2	2	2.7			3	3	6.0			2	2	2.2			0	0	0			0	0	0		
3.6	0	0	0			1	1	2.0			1	1	1.1			2	3	2.4			0	0	0		
3.7	0	0	0			0	0	0			0	0	0			0	0	0			0	0	0		
3.8																0	0	0			0	0	0		

Online Resource 1 Continued, years 1995-1999

Year	1995					1996					1997					1998					1999				
Capture %	1.37					1.99					1.11					1.18					1.52				
ID	r	s	N_C	N_{CR}	SE	r	s	N_C	N_{CR}	SE	r	s	N_C	N_{CR}	SE	r	s	N_C	N_{CR}	SE	r	s	N_C	N_{CR}	SE
3.9	1	1	1.4			0	0	0			0	0	0			0	0	0			0	0	0		
3.10																0	0	0			0	0	0		
3.11																0	0	0			0	0	0		
3.12	0	0	0			0	0	0			0	0	0			0	0	0			0	0	0		
3.13																0	0	0			0	0	0		
3.14																									
3.15																									
3.16																									
4.1	24	38		37.9	7.2	8	10	15.9			0	0	0			1	3	1.2			8	31	12.1		
4.2	0	0	0			0	0	0			0	0	0			0	0	0			0	0	0		
4.3	0	0	0			0	0	0			0	0	0			0	0	0			0	0	0		
4.4	2	3	2.7			0	0	0			0	0	0			2	2	2.4			2	2	3.0		
4.5	1	1	1.4			1	1	2.0			0	0	0			0	0	0			0	0	0		
4.6																0	0	0			0	0	0		
4.8																1	4	1.2			0	0	0		
4.9																1	14	1.2			0	0	0		
4.10																0	0	0			0	0	0		
4.11																0	0	0			0	0	0		
4.12																0	0	0			1	1	1.5		
4.13																									
5.1	1	1	1.4			1	2	2.0			0	0	0			0	0	0			0	0	0		
5.2	0	0	0			0	0	0			0	0	0			0	0	0			0	0	0		
5.3	3	7	4.1			0	0	0			1	1	1.1			0	0	0			2	2	3.0		
5.4																3	11	3.5			0	0	0		
7.1																					0	0	0		
7.2																					1	1	1.5		

Online Resource 1 Continued, years 1995-1999

Year	1995					1996					1997					1998					1999				
Capture %	1.37					1.99					1.11					1.18					1.52				
ID	r	s	N_C	N_{CR}	SE	r	s	N_C	N_{CR}	SE	r	s	N_C	N_{CR}	SE	r	s	N_C	N_{CR}	SE	r	s	N_C	N_{CR}	SE
7.3																					1	1	1.5		
7.4																					0	0	0		
7.5																0	0	0			0	0	0		2.4
7.6																0	0	0			0	0	0		
7.7																2	4	2.4			15	32		18.1	
7.8																1	1	1.2			0	0	0		
7.9																0	0	0			0	0	0		
7.10																0	0	0			0	0	0		
7.11																0	0	0			0	0	0		
7.12																1	2	1.2			0	0	0		
7.13																					0	0	0		
8.1																2	2	2.4			0	0	0		
8.2																2	2	2.4			3	3	4.5		
8.5																0	0	0			0	1	0		
8.6																0	0	0			0	0	0		
8.7																0	0	0			0	0	0		
8.8																0	0	0			5	6	7.6		
8.9																3	4	3.5			0	0	0		
8.10																0	0	0			0	0	0		
8.11																3	4	3.5			0	0	0		
8.12																0	0	0			0	0	0		13.2
8.13																0	0	0			0	0	0		
9.2																0	0	0			8	11	12.1		
9.3																11	16		19.9	6.8	18	24		39.6	
9.4																0	0	0			0	0	0		
9.5																7	7	8.3			7	7	10.6		

Online Resource 1 Continued, years 1995-1999

Year	1995					1996					1997					1998					1999				
Catchability	1.37					1.99					1.11					1.18					1.52				
ID	r	s	N_C	N_{CR}	SE	r	s	N_C	N_{CR}	SE	r	s	N_C	N_{CR}	SE	r	s	N_C	N_{CR}	SE	r	s	N_C	N_{CR}	SE
9.6																2	5	2.4			1	1	1.5		
9.7																0	0	0			0	0	0		
9.8																6	9	7.1			8	15	12.1		
9.9																2	4	2.4			1	1	1.5		
9.10																					8	11	12.1		
9.11																									
9.12																									

Online Resource 1 Continued, years 2000-2006

Year	2000					2001					2002					2005					2006				
Capture %	1.30					1.66					2.66					1.26					1.84				
ID	r	s	N_C	N_{CR}	SE	r	s	N_C	N_{CR}	SE	r	s	N_C	N_{CR}	SE	r	s	N_C	N_{CR}	SE	r	s	N_C	N_{CR}	SE
1.1	0	0	0			0	0	0																	
1.2	12	15		32.3	16.0	3	3	5.0																	
1.3	0	0	0			0	0	0			1	2	2.7			0	0	0							
1.4	5	6	6.5			3	7	5.0			2	2	5.3			0	0	0			5	6	9.2		
1.5	0	0	0			0	0	0								0	0	0							
1.6	0	0	0			1	1	1.7			1	1	2.7			0	0	0			0	0	0		
1.7	18	46		20	1.7	3	4	5.0			3	4	8.0								5	7	9.2		
1.8	0	0	0			0	0	0			0	0	0			10	11	12.6			0	0	0		
1.9	9	12	11.7			5	5	8.3			2	2	5.3								0	0	0		
1.10	0	0	0			0	0	0								0	0	0			0	0	0		
1.11	0	0	0			0	0	0			1	1	2.7			0	0	0			0	0	0		
1.13																									
2.1	60	90		102.9	14.0	39	68		54.9	6.73	42	80		54.6	5.4	7	22	8.8			9	18	16.5		
2.2	2	2	2.6			0	0	0																	
2.3	0	0	0			0	0	0																	
2.4	0	0	0			0	0	0			0	0	0												
3.1	14	17		42.3	21.4	6	7	9.9			0	0	0			5	7	6.3			1	1	1.8		
3.2	12	18		20.6	6.3	6	12	9.9			4	6	10.6			7	23	8.8			12	13		80.1	75.9
3.3	13	66		13.1	0.3	1	1	1.7			13	30		15.1	1.9	14	44		14.7	1.0	22	76		22.8	1.0
3.4	9	37	11.7			8	10	13.3			1	1	2.7			3	4	3.8			7	7	12.9		
3.5	1	1	1.3			0	0	0			8	11	21.3			0	0	0			0	0	0		
3.6	0	0	0			0	0	0			1	1	2.7			1	1	1.3			0	0	0		
3.7	0	0	0			0	0	0			0	0	0			0	0	0			0	0	0		
3.8	0	0	0			2	4	3.3			0	0	0			5	23	6.3			10	23	18.4		

Online Resource 1 Continued, years 2000-2006

Year	2000					2001					2002					2005					2006				
Capture %	1.30					1.66					2.66					1.26					1.84				
ID	r	s	N_C	N_{CR}	SE	r	s	N_C	N_{CR}	SE	r	s	N_C	N_{CR}	SE	r	s	N_C	N_{CR}	SE	r	s	N_C	N_{CR}	SE
3.9	0	0	0			0	0	0			0	0	0												
3.10	0	0	0			0	0	0			0	0	0												
3.11	1	2	1.3			0	0	0			0	0	0												
3.12	0	0	0			0	0	0			0	0	0												
3.13	0	0	0			0	0	0			0	0	0												
3.14																									
3.15																									
3.16																									
4.1	5	11	6.5			4	9	6.6			1	2	2.7												
4.2	0	0	0			0	0	0			1	1	2.7												
4.3	0	0	0			0	0	0			0	0	0												
4.4	0	0	0			0	0	0			0	0	0												
4.5	0	0	0			0	0	0			0	0	0												
4.6	1	1	1.3			0	0	0			0	0	0												
4.8	0	0	0			0	0	0			0	0	0			0	0	0			0	0	0		
4.9	0	0	0			3	6	5.0			2	2	5.3			4	8	5.0			5	5	9.2		
4.10	0	0	0			0	0	0			0	0	0												
4.11	0	0	0			0	0	0			1	1	2.7												
4.12	0	0	0			0	0	0			0	0	0												
4.13																									
5.1	0	0	0			0	0	0																	
5.2	0	0	0			0	0	0																	
5.3	0	0	0			0	0	0																	
5.4	0	0	0			0	0	0																	
7.1	0	0	0			0	0	0			0	0	0												
7.2	1	3	1.3			0	0	0			3	5	8.0			3	3	3.8			14	28		17.6	2.8

Online Resource 1 Continued, years 2000-2006

Year	2000					2001					2002					2005					2006				
Capture %	1.30					1.66					2.66					1.26					1.84				
ID	r	s	N_C	N_{CR}	SE	r	s	N_C	N_{CR}	SE	r	s	N_C	N_{CR}	SE	r	s	N_C	N_{CR}	SE	r	s	N_C	N_{CR}	SE
7.3	0	0	0			0	0	0			5	6	13.3			0	0	0			25	35		48.9	12.2
7.4	0	0	0			0	0	0			0	0	0												
7.5	0	0	0			0	0	0			0	0	0												
7.6	0	0	0			8	12	13.3								7	13	8.8							
7.7	12	30		13.4	1.5	6	12	9.9			25	41		37.7	6.6						14	20		48.9	15.5
7.8	0	0	0																						
7.9	0	0	0			6	12	9.9			0	0	0			2	2	2.5			8	19	14.7		
7.10	0	0	0			0	0	0			0	0	0												
7.11	0	0	0			0	0	0			0	0	0												
7.12	0	0	0								0	0	0			0	0	0			7	7	12.9		
7.13	0	0	0			0	0	0			0	0	0			0	0	0			0	0	0		
8.1		0	0			0	0	0																	
8.2	0	0	0			1	1	1.7																	
8.5	0	0	0			0	0	0																	
8.6	0	0	0			0	0	0																	
8.7	0	0	0			0	0	0																	
8.8	2	3	2.6			0	0	0																	
8.9	5	10	6.5			1	1	1.7																	
8.10	0	0	0			0	0	0																	
8.11	3	4	3.9			2	2	3.3																	
8.12	1	1	1.3			2	3	3.3																	
8.13	0	0	0			0	0	0																	
9.2	2	2	2.6								6	6	15.9			0	0	0			18	24		39.6	13.2
9.3	11	20		14.9	3.2						3	3	8.0			0	0	0			1	1	1.8		
9.4	0	0	0			0	0	0			0	0	0			0	0	0			0	0	0		

Online Resource 1 Continued, years 2000-2006

Year	2000					2001					2002					2005					2006				
Capture %	1.30					1.66					2.66					1.26					1.84				
ID	r	s	N_C	N_{CR}	SE	r	s	N_C	N_{CR}	SE	r	s	N_C	N_{CR}	SE	r	s	N_C	N_{CR}	SE	r	s	N_C	N_{CR}	SE
9.5	1	1	1.3								15	18	39.9	47.8	24.3	5	5	6.3			8	8	14.7		
9.6	0	0	0			0	0	0																	
9.7	0	0	0			0	0	0			0	0	0												
9.8	17	27		26.7	6.0	10	13	16.6			24	41	63.8	34.5	5.6	19	34		26.1	4.4	34	65		44.1	4.8
9.9	1	1	1.3			0	0	0			0	0	0			2	2	2.5			6	6	11.0		
9.10	7	10	9.1			9	13	14.9			9	12	23.9			6	10	7.5			5	5	9.2		
9.11	0	0	0																						
9.12																0	0	0			11	14		27.8	13.6

Online Resource 1 Continued, years 2008-2019

Year	2008					2015					2019				
Capture %	1.16					4.10					2.35				
ID	r	s	N_C	N_{CR}	SE	r	s	N_C	N_{CR}	SE	r	s	N_C	N_{CR}	SE
1.1	0	0	0			3	3	12			0	0	0		
1.2						0	0	0							
1.3	0	0	0			0	0	0							
1.4						2	2	8.2			27	42		43.8	8.2
1.5						0	0	0							
1.6						0	0	0							
1.7															
1.8											0	0	0		
1.9															
1.10						3	4	12							
1.11											1	1	2.4		
1.13											4	5	9.4		
2.1	3	3	3.5			0	0	0							
2.2	0	0	0			0	0	0							
2.3	0	0	0			0	0	0			0	0	0		
2.4						0	0	0			0	0	0		
3.1						2	3	8.2			1	1	2.4		
3.2	2	14	2.3												
3.3	11	64		11	0.2										
3.4	10	19	11.6			3	3	12			10	18	23.5		
3.5						0	0	0							
3.6															
3.7	0	0	0			0	0	0			6	8	14.1		
3.8	8	11	9.2								20	47		23	2.2
3.9	0	0	0												
3.10	0	0	0												
3.11	0	0	0			0	0	0			0	0	0		
3.12	0	0	0								0	0	0		
3.13	0	0	0								0	0	0		
3.14						0	0	0							
3.15											0	0	0		
3.16											0	0	0		
4.1															
4.2															
4.3	0	0	0												
4.4	0	0	0			0	0	0							
4.5	0	0	0			0	0	0							
4.6						0	0	0			0	0	0		
4.8						0	0	0			0	0	0		
4.9											6	6	14.1		
4.10	0	0	0								1	1	2.4		

Online Resource 1 Continued, years 2008-2019

Year	2008					2015					2019				
Capture %	1.16					4.10					2.35				
ID	r	s	N_C	N_{CR}	SE	r	s	N_C	N_{CR}	SE	r	s	N_C	N_{CR}	SE
4.11	0	0	0			0	0	0			0	0	0		
4.12	0	0	0			1	1	4.1			0	0	0		
4.13						1	2	4.1			0	0	0		
5.1						0	0	0							
5.2						0	0	0			0	0	0		
5.3						0	0	0			4	4	9.4		
5.4						0	0	0							
7.1	0	0	0			0	0	0			0	0	0		
7.2															
7.3						0	0	0							
7.4															
7.5						0	0	0							
7.6	0	0	0								0	0	0		
7.7						7	8	29			20	29		36.5	9.3
7.8											0	0	0		
7.9															
7.10															
7.11						0	0	0			0	0	0		
7.12											0	0	0		
7.13						0	0	0			1	1	2.4		
8.1						0	0	0			0	0	0		
8.2						7	7	29			1	1	2.4		
8.5															
8.6						0	0	0			0	0	0		
8.7															
8.8											19	37		24.3	3.4
8.9															
8.10						0	0	0			0	0	0		
8.11						5	6	21							
8.12						4	5	16			1	1	2.4		
8.13											0	0	0		
9.2	5	14	5.8			1	1	4.1			5	7	11.8		
9.3						0	0	0			0	0	0		
9.4						0	0	0			0	0	0		
9.5						25	41		37.7	6.6	33	42		83.4	23.5
9.6															
9.7						0	0	0							
9.8	27	50		35.9	4.7	29	60		35.6	3.6	71	187		78.1	3.2
9.9						0	0	0			1	1	2.4		
9.10	10	27	11.6			2	2	8.2			1	1	2.4		
9.11						13	22		18.9	4.3	33	59		45.3	5.7
9.12											1	1	2.4		
