# Supplementary material for: Metapopulation dynamics over 25 years of a beetle, Osmoderma eremita, inhabiting hollow oaks
Source: Oecologia. 2020 Nov 7;194(4):771–80. doi: 10.1007/s00442-020-04794-7 (PMC7683440; doi:10.1007/s00442-020-04794-7)
Supplement: Supplementary file 2 — Supplementary file2 (PDF 447 KB) [file 442_2020_4794_MOESM2_ESM.pdf]

Online Resource 2

Journal: Oecologia

Title: Metapopulation dynamics over 25 years of a beetle, *Osmoderma eremita*, inhabiting hollow oaks

Authors: Ly Lindman, Mattias C. Larsson, Kajsa Mellbrand, Glenn P. Svensson, Jonas Hedin, Olov Tranberg, Thomas Ranius

Corresponding author: L. Lindman, e-mail: Ly.Lindman@slu.s

Online Resource 2 Plausible candidate models (ΔAIC < 2) explaining colonisations and extinctions of *O. eremita* observing colonisations and extinctions by comparing presence/absence over one and two sequential sampling years at the time. Tree ID as a random factor is included in all models. Models are ranked according to their second-order Akaike's information criterion (AIC_c). Sample size (*N*), number of parameters (*k*), model weight (w_i) and McFadden´s R^2 are reported

	N	Int.	Parameter estimates								k	LogLik	ΔAICc	w_i	R^2_{MF}
			Connec-tivity	Alive/ dead	Wood mould volume	Entr. size	Entr. height	Dia-meter	Tree age	No of possible occ.					
1. Colonisation															
a) 1 year	64														
		1.74	0.035							-0.48	4	-31.94	0.00	0.34	0.27
		1.45		0.626						-0.47	4	-32.53	1.19	0.19	0.25
		1.17	0.037	0.667						-0.47	5	-31.45	1.37	0.17	0.28
		1.74								-0.47	4	-32.70	1.51	0.16	0.25
		1.68						0.002		-0.47	4	-32.93	1.98	0.13	0.24
b) 2 years	51														
		0.60	0.029							-0.35	4	-27.27	0.00	0.25	0.12
		0.32		0.472						-0.33	4	-27.70	0.87	0.16	0.10
		0.68					0.001			-0.36	4	-27.75	0.96	0.16	0.10
		0.88			-0.003					-0.34	4	-27.82	1.10	0.15	0.10
		0.30						0.003		-0.34	4	-27.83	1.12	0.14	0.10
		0.77				-0.001				-0.34	4	-27.88	1.22	0.14	0.10

Online Resource 2 Continued

	N	Int.	Connec-tivity	Alive/dead	Wood mould volume	Entr. size	Entr. height	Dia-meter	Tree age	No of possible occ.	k	LogLik	ΔAICc	w_i	R^2_{MF}
			Parameter estimates												
2. Extinction															
b) 1 year	25														
		0.24									2	-17.15	0.00	0.26	
		3.59							-0.007	-0.20	4	-14.67	0.49	0.20	0.14
		2.88						0.02	-0.013	-0.19	5	-13.22	0.76	0.18	0.23
		1.36			-0.007					-0.18	4	-15.12	1.40	0.13	0.12
		1.73		-0.687						-0.2	4	-15.18	1.52	0.12	0.11
		0.89					0.002			-0.21	4	-15.19	1.54	0.12	0.11
a) 2 years	23														
		-2.1						0.02		-0.84	4	-5.30	0.00	0.32	0.50
		-0.7				0.028				-0.5	4	-5.63	0.65	0.23	0.47
		0.90			-0.009					-0.74	4	-5.87	1.13	0.28	0.45
		0.11	0.034							-0.67	4	-6.00	1.38	0.16	0.44
		-1.1						0.03	-0.005	-0.97	5	-4.64	1.98	0.12	0.56
